# Practical Guide to Large Language Models for Information Extraction in Behavioral Health Notes: Tutorial

**DOI:** 10.2196/97498

**Published:** 2026-09-24

**Authors:** Diya Saha, Juliet B Edgcomb

**Affiliations:** 1 Department of Computer Science, University of California, Irvine, 6210 Donald Bren Hall, Irvine, CA, 92697, United States, 1 9498247427; 2 Center for Community Health, UCLA Mental Health Informatics & Data Science (MINDS) Hub, University of California, Los Angeles, Los Angeles, CA, United States

**Keywords:** large language models, clinical notes, electronic health records, suicide risk, medication adherence, natural language processing, structured outputs, zero-shot learning, information extraction

## Abstract

**Background:**

Mental health clinical notes contain decision-critical information often absent from structured electronic health record fields. Large language models (LLMs) can extract clinically relevant signals from narrative text; however, variability in output format, limited reproducibility, and inconsistent evaluation remain barriers to clinical deployment. Despite rapid advances in LLM-based information extraction, clear and reproducible guidance for interdisciplinary clinical teams is limited.

**Objective:**

This tutorial aims to present a structured workflow for zero-shot information extraction from mental health clinical notes using locally deployed open-source LLMs. It aims to reduce barriers for clinicians and researchers with limited familiarity with natural language processing (NLP) or LLM-based pipelines. Each stage includes key decision points and examples. The workflow is illustrated on two tasks using synthetic notes: (1) detection of self-injurious thoughts and behaviors (SITB) in pediatric emergency department (ED) notes and (2) antipsychotic medication nonadherence detection in outpatient notes, using schema-constrained outputs and standardized evaluation.

**Methods:**

We describe a five-stage zero-shot LLM pipeline: (1) infrastructure setup with local deployment via *Ollama* to prevent protected health information (PHI) transmission; (2) task definition specifying the clinical construct, output format, and evaluation; (3) dataset preparation using synthetic notes; (4) iterative prompt development using a hold-out development set with binary and Likert scale outputs constrained via JSON schemas; and (5) output parsing, normalization, and validation. We generated 300 synthetic notes per task using separate LLMs for generation and evaluation; 200 notes were used for evaluation, and 100 notes (50 positive and 50 negative) were used as a prompt-development set and excluded from final metrics. Evaluation used Large Language Model Meta AI (Llama) 3.2 and Llama 3.3 with deterministic decoding (temperature=0). Performance was assessed using accuracy, precision, recall, and *F*_1_-score; Likert thresholds were optimized using the Youden index with bootstrapped CIs.

**Results:**

We demonstrated the pipeline’s functionality using 2 example behavioral health detection tasks. Across both examples, the more capable model (Llama 3.3) performed better than the lighter model used earlier in development (Llama 3.2), and we described how the pipeline’s evaluation and error-analysis steps work in practice. These examples also illustrated 2 useful design choices: requiring the model to output in a fixed format reduced errors, and using a graded rating scale, rather than a simple yes/no format, allowed the detection threshold to be adjusted based on clinical risk tolerance. These results are meant to show that the pipeline works as intended, not to serve as a benchmark of real-world accuracy.

**Conclusions:**

A schema-driven, zero-shot LLM workflow can support reproducible extraction of clinically relevant information from narrative notes. Local deployment enables processing without transmitting PHI to external servers. This tutorial provides a transferable methodology for institutional adaptation and validation prior to clinical use. All prompts, code, and datasets are publicly available via Zenodo (European Organization for Nuclear Research [CERN]).

## Introduction

Mental health clinical notes contain decision-critical information not captured in structured fields [1]. Note documentation includes safety appraisals, psychosocial context, and mediators of treatment response (eg, adherence) [2,3]. However, free-text storage limits reliable signal identification at scale, and manual chart review cannot meet this demand [4]. Large language models (LLMs) increasingly address this gap, supporting medical record summarization, information extraction, and reasoning over clinical text. Mental health clinicians now often interact with LLMs to assist with triage support, risk stratification, and chart review [5,6]. Yet rigorous clinical validation lags behind adoption [7,8]. Proprietary software and free-form text outputs of LLMs complicate validation, hinder downstream analytics [9,10], and limit reproducibility. For clinicians and health systems to understand and use LLM-based systems safely, transparent pipelines (ie, those that define inputs, outputs, validation rules, and failure modes) are essential.

Unlike specialties where risk is captured in structured, coded fields, mental health documentation relies heavily on narrative text to convey safety-critical information, which is frequently expressed through indirect, negated, or context-dependent language, increasing both the extraction burden and the consequences of extraction errors for risk stratification and treatment decisions.

Researchers have applied both general-purpose models (eg, GPT family models and Llama variants) and domain-adapted clinical LLMs (eg, the Bidirectional Encoder Representations from Transformers [BERT]–based models Clinical BERT [ClinicalBERT] and Biomedical and Clinical BERT [BioClinicalBERT]) to free-text psychiatric documentation [11] and have successfully detected suicidal ideation, nonsuicidal self-injury, depression severity, substance use, and medication adherence [5]. Despite these advances, performance will always vary across tasks and documentation styles. Models may overweight explicit negation, conflate nuanced symptoms, or misinterpret psychosocial contexts. Clear, reproducible guidance for interdisciplinary clinical teams remains limited [7], even as computer scientists and natural language processing (NLP) experts refine evaluation frameworks and fine-tuning strategies [12]. A structured tutorial that defines schemas (ie, predefined rules specifying the exact fields and format an LLM’s output must contain), prompting constraints, validation logic, and performance metrics can therefore help bridge the gap between technical advances and practical clinical use.

This tutorial presents an end-to-end workflow for extracting clinically relevant information from notes using open-source out-of-the-box LLMs. We apply the same pipeline to two illustrative examples: (1) detection of self-injurious thoughts and behaviors (SITBs) among youth presenting to the emergency department (ED) with mental health concerns; and (2) antipsychotic medication nonadherence detection in adults with serious mental illness.

## Methods

### Step 1: Setup Initial Workflow

#### Setup Overview

**Figure 1.**
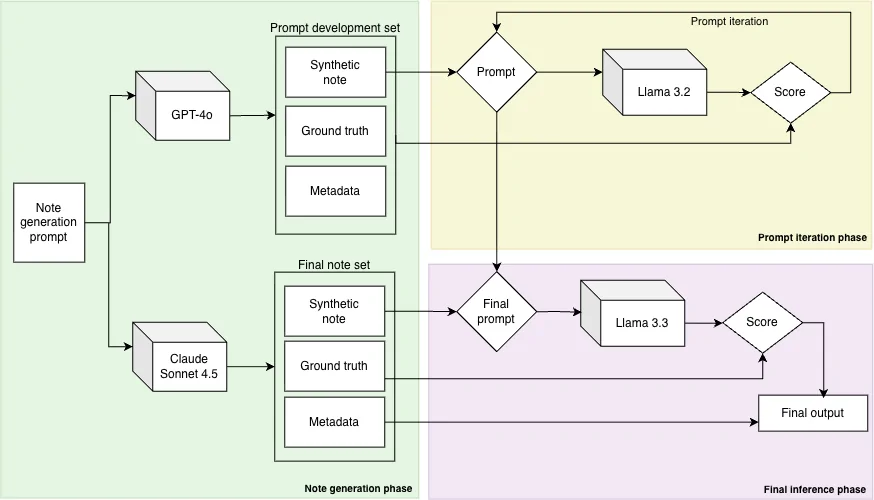
Workflow for synthetic note generation, prompt iteration, and final evaluation. In the note generation phase (green), a shared note generation prompt is used to independently produce 2 synthetic datasets: a prompt-development set generated by GPT-4o and a final note set generated by Claude Sonnet 4.5, each consisting of a synthetic clinical note, an associated ground-truth label, and metadata. In the prompt iteration phase (yellow), the development set is used to refine the extraction prompt. The prompt is applied to Llama 3.2, its output is scored against the corresponding ground truth, and this score is fed back to update the prompt across iterations until performance converges. The finalized prompt then carries forward as the final prompt into the final inference phase (purple), where it is applied together with the final note set to Llama 3.3. The resulting output is scored against ground truth, and the score and metadata are combined to produce the final output.

Extracting clinical information from notes requires a reproducible and auditable workflow. Unlike traditional rule-based systems, LLMs can produce variable outputs depending on inference parameters, prompt phrasing, and model stochasticity (ie, the tendency of generative models to produce different outputs for the same input across repeated runs). Without careful workflow design, repeated runs on the same clinical text may yield different outputs, complicate evaluation, and limit clinical reliability. The initial workflow presented here is therefore designed to promote reproducibility by controlling sources of variability across prompt design, inference configuration, output structure, and evaluation procedures. The overall structure of this workflow is illustrated in Figure 1, which uses distinct generator models for development (GPT) and final testing (Claude Sonnet 4.5) to ensure that the prompt is validated on note styles and model behaviors on which it was not tuned, thereby reducing the risk of overfitting to a single generator’s specific patterns.

#### Setup Infrastructure

The classification workflow begins with establishing a secure computing environment designed to support consistent and reproducible results. The appropriate deployment strategy depends on institutional policy, computational resources, and regulatory constraints. A key advantage of open-source LLMs is that they can be deployed entirely within institutional computing environments, allowing clinical text to remain inside secure systems. Local inference reduces reliance on external APIs and avoids transmitting protected health information (PHI) to third-party servers. Institutions may also implement inference using secure on-premises GPU clusters or Health Insurance Portability and Accountability Act (HIPAA)–compliant cloud environments that provide contractual privacy guarantees. For behavioral health notes specifically, this issue is compounded by additional regulatory sensitivity. Substance use disorder treatment information carries separate federal confidentiality protections (42 Code of Federal Regulations [CFR] Part 2) beyond standard HIPAA safeguards, and psychiatric documentation carries elevated reputational and legal risks if exposed, reinforcing the case for local, non-API deployment.

As LLM behavior can vary depending on system configuration, reproducibility requires careful documentation of the parameters that control how the model generates output. These parameters together define the inference configuration and should remain consistent during development and evaluation of the workflow. At minimum, the following elements must be documented: (1) model version and quantization settings, where quantization refers to reducing the numerical precision of a model’s weights (eg, from 16-bit to 4-bit) to shrink memory footprints and speed up inference, typically with a small accuracy trade-off (eg, Llama-3.1-8B-Instruct at 4-bit GPTQ quantization); (2) hardware configuration (eg, central processing unit (CPU), graphics processing unit (GPU), available memory, and cluster or multi-GPU configuration if applicable); (3) decoding parameters (eg, temperature and maximum tokens); (4) processing strategy (batch vs sequential inference); and (5) logging and error-handling procedures.

Deterministic decoding is particularly important when model outputs are evaluated programmatically. It refers to an inference configuration in which outputs are fully reproducible given the same input [13]. Among generation parameters, temperature controls randomness; setting temperature=0 enforces deterministic decoding and ensures identical outputs across runs (Table 1).

**Table 1.** Summary of runtime (in seconds) across the models and tasks (pediatric SITB^a^ and adult antipsychotic medication nonadherence detection)*.*

Model name	Task A (pediatric SITB)	Task B (adult antipsychotic medication nonadherence)	
	Likert^b^	Likert	Binary
Llama 3.2 (3 B^d^)	423.39 s^e^	404.43 s	221.69 s
Llama 3.3 (70 B)	1515.37 s	1527.82 s	1105.00 s

a SITB: self-injurious thoughts and behaviors.

b Likert: Likert-scale prompting.

c Binary: binary prompting configuration.

d B: billion parameters.

e Runtime values are reported in seconds.

#### Define the Problem

With setup infrastructure in place, the next step is to clearly define the classification task. LLMs can support many types of clinical applications, including information extraction, summarization, and classification. Here, we focus on prompt-based classification, in which a prompt instructs the LLM to analyze clinical text and assign a structured prediction based on predefined criteria. Effective prompt-based classification requires a well-defined 3-part objective that specifies (1) the clinical construct of interest: the concept to be detected, specified in concrete, measurable terms to enable consistent labeling (eg, presence of a condition, behavioral risk, or severity); (2) the target output format: how predictions are expressed (eg, binary, ordinal, or multiclass), which determines prompt design and ground-truth label structure; and (3) the evaluation framework: how predictions are assessed against reference labels, including metric selection and alignment procedures.

In mental health contexts, target constructs are rarely confirmed by an objective test or laboratory value; they must instead be inferred from a patient’s reported symptoms, behavior, and clinician interpretation as recorded in narrative text, making precise construct definition especially important to avoid drift between what the label is meant to capture and what the prompt actually elicits.

#### Gather Notes

Each synthetic note was generated from a structured specification sheet defining required fields, narrative tone, and label-conditional constraints, with ground-truth labels embedded during generation and stored separately from the note text. To reduce the risk of generation-evaluation overlap, 2 separate language models were used, one to generate a preliminary prompt-development set and a separate model to generate the final evaluation corpus, with positive and negative cases generated in separate batches to minimize label contamination. Full methodological detail, including the generation prompt structure, schema validation procedure, and safeguards against templated or duplicated text, is provided in Multimedia Appendix 1, with the full example specification sheet in Multimedia Appendix 2.

#### Create a Baseline Prompt

With the dataset prepared, the next step is to instruct the model to perform the classification task. In prompt-based clinical classification, the LLM acts as a guided classifier by interpreting clinical text and assigning output based on instructions provided in the prompt. This approach relies on carefully written instructions to direct the model behavior. Because the model itself is not retrained (as in fine-tuning), the prompt must clearly state the task definition and output requirements. Prompt wording substantially influences classification behavior and output formatting [13]. Minor phrasing changes can alter interpretation of ambiguous clinical language and may introduce formatting errors that interfere with structured output parsing. A structured prompt-development process is therefore necessary before final evaluation.

The prompt development set should be reserved exclusively for prompt iteration and excluded from final performance reporting. Prompt iteration should begin with a baseline prompt derived directly from the task definition. At minimum, the prompt should define the clinical role assigned to the model, explicit classification criteria (eg, positioning the model as a clinical documentation reviewer screening for risk-relevant content, rather than a general-purpose assistant), and the expected output format (eg, binary label, ordinal score, or structured schema). Subsequent refinements should be guided by observed failure patterns. We discuss refinement in detail in Step 2.

#### Prompt Output Formats

Prompts may request model predictions in several different formats depending on the task design. Common approaches include binary classification labels, multiclass labels, ordinal rating scales, or probabilistic outputs. The appropriate format depends on the clinical objective and the structure of the reference labels. One commonly used format is binary prompting, in which the model returns a categorical classification indicating whether the target condition is present or absent (eg, yes/no). Binary prompting allows predictions to be directly compared with ground-truth labels and supports standard classification metrics such as accuracy, precision, recall, and *F*_1_-score. In some tasks, however, clinical evidence may be distributed throughout the note and may not correspond neatly to a strict binary decision. In such cases, an ordinal prompting format can capture varying degrees of confidence in the model’s assessment. One such ordinal format is the Likert score. In Likert prompting, the model returns an ordinal score reflecting the strength of evidence that the target condition is present. In our implementation, the model produced a discrete score s∈ {−3, −2, −1, 0, 1, 2, 3}, where negative values indicate evidence against the condition and positive values indicate evidence supporting the condition. This is particularly relevant in mental health documentation, where risk is often graded rather than categorical; a note describing resolved, historical suicidal ideation and one describing an active plan both refer to the same construct but carry meaningfully different clinical weight that a strict binary label can flatten.

Ordinal responses can capture degrees of uncertainty in clinical language that may not be fully represented by binary labels. Likert prompting can therefore provide additional insight into borderline or ambiguous cases while still allowing conversion to binary predictions during evaluation. Other prompting strategies are also possible. For example, prompts may request probability estimates, multilabel outputs, or structured clinical categorizations. The choice of output format should be guided by the clinical question, dataset labeling scheme, and evaluation methodology.

#### Parse and Normalize Model Outputs

Once prompts have been executed and model predictions generated, the next step is to extract and prepare these outputs for the evaluation framework. This process involves output parsing, schema validation, output normalization, label alignment, and integrity checks.

##### Output Parsing

Parsing refers to the process of reading the model’s raw output and extracting the prediction required for evaluation. In many workflows, this involves identifying a specific field within a structured output format (eg, a JSON field containing the predicted label). Because evaluation requires alignment between predicted labels and reference labels, the parsing step must reliably extract the relevant prediction from the model response.

##### Schema Validation

If the LLM was instructed to return results in a specific format, the first step during parsing is to verify that the output conforms to the expected schema. Responses that are malformed or incorrectly structured outputs (ie, schema violations) or contain additional text outside the expected format should be flagged before any further processing. Any outputs that cannot be reliably parsed or validated should be flagged for additional review rather than passed silently to downstream steps.

##### Output Normalization

Once predictions have been extracted, normalization is applied to standardize the parsed outputs before comparison with ground-truth labels. Even when prompts request a strict output schema, LLM responses may contain small formatting deviations such as additional explanatory text, leading or trailing whitespace, inconsistent capitalization, or minor variations in field formatting. Because evaluation scripts typically rely on exact string matching, these inconsistencies can be misinterpreted as classification errors if they are not corrected during preprocessing. Normalization procedures may include trimming leading or trailing whitespace, standardizing capitalization (eg, converting labels to lowercase), mapping semantically equivalent variants to a canonical label (eg, “yes,” “Yes,” and “YES” → “yes”), removing unintended commentary or markdown artifacts, and validating and reformatting minor JSON inconsistencies when possible.

Normalization should be limited to formatting adjustments and should not modify the semantic content of the prediction. For example, trimming whitespace or adjusting capitalization (eg, “Schizophrenia” → “schizophrenia”) is acceptable, whereas collapsing distinct diagnostic terms (eg, “Simple Schizophrenia” → “schizophrenia”) would alter the meaning of the prediction and should not be performed. Any output that cannot be reliably parsed or normalized should be flagged for additional review.

##### Label Alignment and Integrity Checks

After normalization, predicted labels are paired with ground-truth labels using the unique identifiers established during dataset preparation. Only then should performance metrics be computed. Final integrity checks should confirm that each note has exactly one valid predicted label and that all predictions fall within the predefined classification categories for the task. Any missing or invalid outputs identified during the parsing stage should be documented and handled consistently (eg, excluded or counted as errors according to a predefined policy). These safeguards ensure that performance estimates reflect true classification behavior rather than formatting artifacts.

### Evaluate

After predicted labels have been parsed, normalized, aligned with ground-truth labels, and validated through final integrity checks, classification performance can be quantified.

The optimal choice of performance metrics depends on the type of classification task being performed. For binary classification tasks, commonly reported measures include accuracy, precision, recall (sensitivity), specificity, and *F*_1_-score. Precision (positive predictive value) reflects how trustworthy a positive prediction is: of all notes the pipeline flags as positive, what proportion are truly positive, with low precision indicating frequent false alarms. Recall (sensitivity) reflects how completely the pipeline captures true cases: of all notes that are truly positive, what proportion the pipeline correctly identifies, with low recall indicating missed cases. The *F*_1_-score summarizes precision and recall into a single value. This is useful when both error types carry meaningful cost. The appropriate emphasis depends on the clinical consequences of each error type. Recall may be prioritized in safety-critical mental health detection tasks such as suicide risk screening, where missing a true positive case could delay identification of a patient in crisis, whereas a higher precision threshold may be preferred in lower-acuity screening tasks to limit clinician alert burden. The metrics should include 95% CIs (eg, bootstrapping with n=1000 resamples), with sample sizes reported for each metric computation.

A confusion matrix may be computed to examine class-level performance by cross-tabulating predicted labels against ground-truth labels to identify true positives, true negatives, false positives, and false negatives. Unlike aggregate accuracy, the confusion matrix reveals systematic error patterns and indicates which direction prompt refinement should take: a pattern dominated by false negatives suggests that the classification criteria are too strict or insensitive to subtle evidence and should be loosened, while a pattern dominated by false positives suggests criteria are too permissive and should be tightened. Reporting raw counts of misclassified results helps contextualize aggregate performance metrics. This level of detail is particularly important in clinical contexts where different error types may carry unequal consequences. Beyond summary metrics, misclassifications must also be qualitatively reviewed to determine whether errors arise from prompt ambiguity, insufficient task definition, dataset labeling inconsistencies, or genuinely ambiguous clinical documentation. If systematic errors reflect misalignment in earlier stages of the workflow, additional prompt or task refinement may be warranted. If remaining errors primarily reflect true clinical ambiguity rather than problems with the prompt, output format, or evaluation pipeline, the workflow may be considered ready for further refinement.

### Example

All inference experiments were conducted in locally deployed environments using the *Ollama* inference server, so clinical notes were never transmitted to external servers. Two versions of Llama were used at different stages, applied identically across both tasks. Llama 3.2 supported rapid, low-cost prompt iteration, while Llama 3.3 was reserved for final evaluation using the frozen prompts (see Refine the Model). Prompt development ran on a MacBook Pro (Apple M3 Pro, 14-core GPU, 36 GB unified memory) using quantized Llama 3.2 (~2 GB); final evaluation ran on a Mac Studio (Apple M2 Ultra, 24-core CPU, 76-core GPU, 128 GB unified memory) using quantized Llama 3.3:70B (~42 GB). Notes were processed sequentially to approximate realistic deployment conditions; Table 1 summarizes models, tasks, prompting configurations, and runtime.

We demonstrate the workflow on 2 clinically distinct tasks. Task A (pediatric SITB detection) evaluates whether a note documents suicidal ideation, suicide attempts, or nonsuicidal self-injury; Task B (antipsychotic medication nonadherence detection) evaluates whether a note documents nonadherence to prescribed antipsychotic medication in individuals with serious mental illness. Both tasks produce either a binary decision or an ordinal confidence score, which are evaluated against ground-truth labels assigned by constructions during synthetic note generation, using accuracy, precision, recall, and *F*_1_-score for binary outputs, and Youden-optimal thresholding to convert Likert predictions to binary decisions.

**Figure 2.**
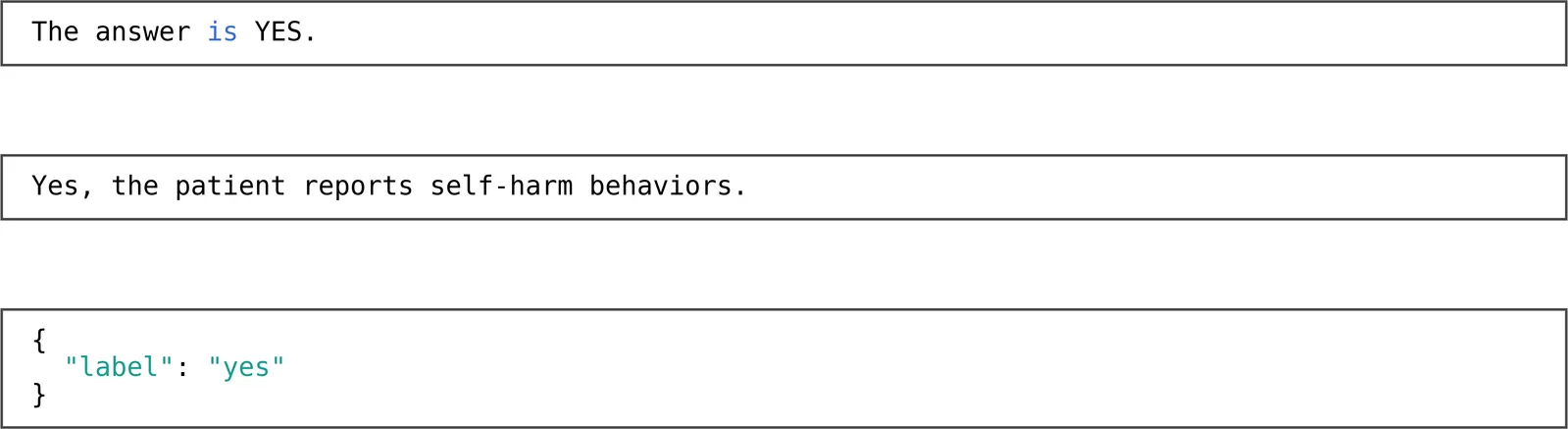
Initial large language model (LLM) outputs with the baseline prompts.

Prompt development began from minimal binary baseline prompts with no additional guidance: “Determine whether the clinical note contains suicidal ideation, suicide attempts, or self-injury” (Task A) and “Determine whether the patient is adherent or nonadherent to prescribed medications” (Task B). These prompts specified no handling of borderline cases, negation, temporality, or output format and initially produced free-form responses with predictions embedded in explanatory text rather than structured labels (Figure 2).

**Figure 3.**
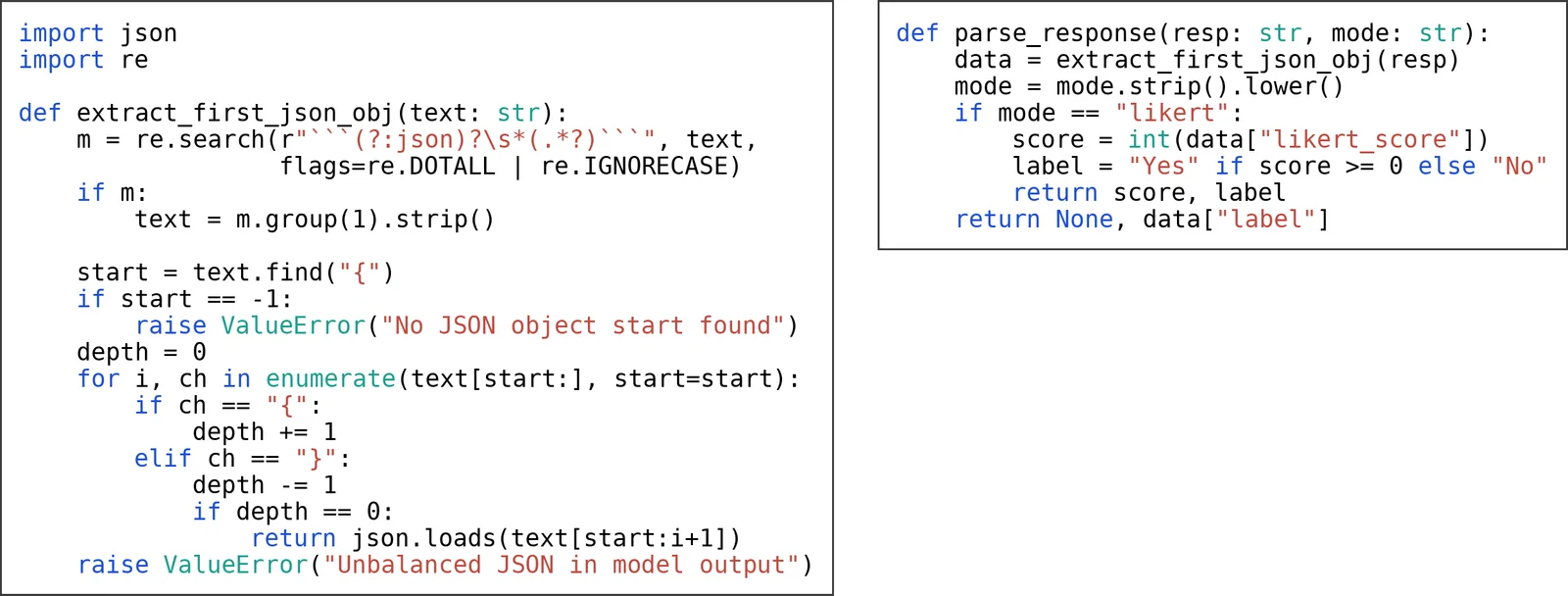
Programmatic extraction and normalization of large language model (LLM)–generated JSON outputs.

Prompts were revised to require structured JSON output, a label field ({yes, no}) for binary prompting, or a discrete score s ∈ {−3,...,3} for Likert prompting, and automated parsing scripts extracted and normalized these fields, excluding (rather than manually correcting) malformed outputs so evaluation reflected model behavior rather than postprocessing (Figure 3). Likert scores were converted to binary predictions using a fixed threshold rule (predict yes if s≥τ), and parsed labels were aligned with ground truth via unique note identifiers.

Classification performance was evaluated using accuracy, precision, recall, and *F*_1_-score, along with confusion matrices to examine class-level performance. Metric priorities differed by task; recall was prioritized for Task A because missing a true SITB-positive case could have significant downstream safety consequences [6], whereas precision carried relatively greater weight for Task B to limit unnecessary clinician follow-up alerts. Misclassified cases were qualitatively reviewed to distinguish errors attributable to ambiguous clinical language, prompt limitations, or label inconsistencies.

### Step 2: Refinement

#### Refinement Overview

The procedures described in Step 1 establish a reproducible baseline classification workflow designed to minimize variability in model outputs. This initial workflow is demonstrated in Figure 1. Initial performance represents a starting point (hereafter referred to as the baseline). Prompt-based clinical classification requires structured, iterative refinement across multiple components of the pipeline. Refinements should be systematic, documented, and evaluated using predefined metrics rather than ad hoc adjustments. Refinements must be tested exclusively on prompt-development sets and never on the final evaluation dataset. This separation prevents implicitly overfitting the techniques to a single dataset and preserves the ability to assess generalization. Refinement may occur at multiple levels of the workflow, including prompt design, output structure, and model selection, a process that is especially iterative for behavioral health text, given its reliance on indirect, negated, and context-dependent language to convey clinically important information.

#### Refine the Prompt

Prompt wording is often an influential factor in LLM classification performance. Because prompt-based workflows rely on natural-language instructions rather than model retraining, even small changes in phrasing can alter how the model interprets ambiguous clinical language, prioritizes evidence, or applies classification criteria. Early modifications to the prompt are ideally minimal and targeted, allowing performance changes to be attributed to specific prompt adjustments rather than rewrites. Several prompting strategies can be used to guide model behavior. Zero-shot prompting provides only task instructions without labeled examples. Few-shot prompting supplements the instructions with example input-output pairs to demonstrate the desired behavior [13,14]. Other techniques, such as chain-of-thought prompting, encourage the model to generate intermediate reasoning steps before producing a final prediction [15]. Each approach can influence how the model processes the task and may affect classification accuracy, reasoning behavior, or output stability. To determine whether refinements meaningfully improve performance, both quantitative metrics and structured qualitative error analysis must be examined after each iteration. These strategies matter more in mental health extraction than in more templated clinical domains, since risk-relevant signal is frequently embedded in hedged or indirect phrasing rather than standardized terminology.

##### Classification Behavior

In early refinement cycles, the primary focus is classification behavior rather than output formatting or structural compliance. Evaluation at this step should pair qualitative assessment with targeted prompt adjustments: (1) are clear positive and negative cases classified correctly? This can be assessed using a 2×2 contingency matrix. Errors on unambiguous cases suggest fundamental task interpretation failures; the prompt should be revised to clarify core classification criteria, (2) how does the model handle borderline or ambiguous language? This can be evaluated through clinician review of false positives and false negatives. If borderline cases are consistently misclassified, inclusion and exclusion criteria should be refined to better define decision boundaries, and (3) do errors reflect systematic misinterpretation? Recurrent error patterns often reveal specific prompt gaps rather than inherent model limitations. Common patterns include overweighting explicit negation (eg, treating “denies suicidal ideation” as overriding documented behavioral evidence); inconsistent temporality handling (eg, classifying historical symptoms as current); and inconsistent decision logic across semantically similar inputs. In behavioral health documentation, these particular error types carry more than cost beyond accuracy; a missed negation or temporality error can misrepresent a patient’s current risk status rather than simply mislabeling a data point. Prompt refinement should directly target whichever patterns are observed.

Refinement at this stage is iterative. Modifications should be incremental and evaluated after each change to isolate the effect of specific prompt adjustments. If performance declines following a modification, the prompt should be reverted to the previous version, and refinement resumed from that point.

##### Improving Consistency and Stability

Once classification performance shows improvement or stabilizes, refinement should also address prediction stability.

Evaluation at this step should include two questions: (1) are semantically similar notes classified consistently? If so, this suggests that the prompt provides sufficiently clear and well-defined classification criteria, enabling the model to apply decision rules consistently across similar inputs; (2) do small variations in phrasing produce unstable predictions? If small linguistic changes result in unstable predictions, this often indicates prompt ambiguity or insufficiently specified classification criteria rather than inherent model randomness.

Prompt refinements aimed at improving stability typically focus on clarifying task instructions and reducing ambiguity in the classification criteria. This may involve restructuring criteria into clearly delineated statements, reinforcing decision boundaries between categories, explicitly requiring the model to select exactly one label, or providing brief guidance before producing the final prediction. Stability testing should be conducted under fixed inference parameters to ensure that any observed variability reflects prompt design rather than sampling effects introduced by stochastic decoding.

##### Output Format Structure

In addition to classification behavior and stability, structural output compliance must be evaluated at each refinement stage. When outputs are processed programmatically, strict output schema adherence is essential for reproducibility. Evaluation at this step should include answering the following: (1) does the model return a valid structured format? (2) Are required fields present and correctly labeled? (3) Does extraneous text or commentary appear outside the schema? and (4) Do formatting artifacts interfere with automated parsing? Persistent formatting errors may reflect prompt ambiguity rather than classification failure. Even when classification accuracy improves, inconsistent structural compliance can compromise downstream evaluation pipelines. Structural compliance can be evaluated by examining whether repeated runs produce valid outputs and whether small variations in input phrasing produce formatting errors or schema violations.

At this stage, four refinement strategies may be considered: (1) strengthen instructions to return only the structured output, for example by explicitly stating that the model should not produce any explanatory text outside the required schema; (2) explicitly prohibit additional commentary, which can reduce responses such as introductory phrases or narrative explanations that appear before or after the structured output; (3) simplify the schema to essential fields, since complex or deeply nested structures may increase the likelihood of formatting errors; and (4) provide a canonical formatting example within the prompt, which gives the model a concrete template for the expected response format. For instance, a prompt may include a generic template such as {“label”: “yes or no,” “reasoning”: “one or 2 sentences explaining the decision”} to illustrate this general pattern. In our implementation, this pattern was adapted for a different purpose; rather than a free-text rationale, the Likert schema required the model to restate its numeric score in words via a likert_score_analysis field (eg, “extremely present” for a score of 3). This functions as a self-consistency check rather than an explanation, because the model must independently articulate what the score means; a mismatch between the numeric score and its stated descriptor may indicate that the numeric value does not reflect the model’s underlying judgment (ie, a potentially hallucinated or unstable score), rather than serving to surface diagnostic reasoning. This field was omitted from the binary output, which was limited to the label alone to minimize parsing complexity.

##### Prompt Finalization

After each revision, the updated prompt should be re-evaluated on the prompt development set. If performance declines, the modification may have introduced unintended ambiguity or shifted the model’s behavior away from the intended task definition. This controlled iteration prevents prompt drift and reduces the risk of overadjustment. Prompt refinement should continue until performance on the prompt-development set stabilizes. A plateau is typically reached when additional prompt modifications no longer produce meaningful improvements in classification metrics, prediction stability, or structural output compliance. At this stage, remaining errors often reflect clinically ambiguous documentation rather than prompt deficiencies. Once performance plateaus and structural output compliance remain consistent across runs, the prompt can be considered sufficiently stabilized and should be frozen. The finalized prompt should then be applied unchanged to the evaluation dataset.

### Refine Model Inputs and Outputs

If prompt refinements fail to fully resolve classification errors, instability, or formatting inconsistencies, another component that can be adjusted is the structure of model inputs and outputs. The way information is presented to the model and the way predictions are returned can influence classification behavior and the reliability of downstream processing.

#### Refining Reasoning Outputs and Response Constraints

Once structural output compliance is stable, an optional refinement is to extend the output schema to include a brief supplementary field alongside the primary classification label. Depending on how it is structured, this field can serve one of 2 distinct purposes. First, a free-text rationale field, most useful for ambiguous or borderline cases, can support error diagnosis and targeted prompt revision by making explicit which evidence in the note drove the prediction. Such fields are diagnostic rather than predictive; they do not directly improve classification performance but can help surface systematic failure patterns, for example, whether the model consistently misinterprets negated statements or underweights relevant sections of a note. Second, for ordinal or Likert-style outputs, a constrained categorical descriptor requiring the model to restate its numeric score in words (eg, “extremely negative” for a score of −3) can instead function as a self-consistency check: because the model must independently articulate what the score means, a mismatch between the numeric score and its stated descriptor can indicate that the numeric value does not reflect the model’s underlying judgment, flagging a potentially hallucinated or unstable prediction rather than explaining it. In either case, the supplementary field should be narrowly scoped, typically one sentence, and placed within the existing schema structure; constraining it to a fixed length reduces variability and prevents it from interfering with downstream parsing.

#### Refining Input Structure

The structure of the input prompt may also influence model performance. When prompts contain multiple types of information, such as clinical notes, contextual metadata, or supporting evidence, organizing these inputs into clearly defined structures can improve model interpretation. Four possible refinements include (1) separating prompt components using labeled sections, (2) embedding contextual information within structured templates, (3) isolating note text using quotation markers or delimiters, and (4) organizing inputs using structured formats such as JSON or XML. Structured inputs may be particularly useful when providing contextual examples or reference data. For example, when a prompt design calls for few-shot examples or input-output pairs, representing these elements as structured JSON objects can clarify both the expected format and the relationships between fields. Similarly, labeling sections within the prompt (eg, Clinical Note, Context, and Instructions) may help the model distinguish between task instructions and source text. Some models may process structured inputs more reliably than unstructured text, particularly when prompts contain multiple sources of information or complex contextual data.

### Refine the Model

#### Model Refinement Overview

When performance plateaus but classification accuracy remains limited, further improvements may require changes beyond prompt design. At this stage, limitations may reflect properties of the underlying language model rather than the prompt itself. Models with larger parameter counts (often referred to as larger-capacity models) may demonstrate stronger instruction adherence, improved contextual reasoning, and more consistent decision behavior [16]. As a result, larger models may improve classification performance in borderline or clinically ambiguous cases and increase prediction stability across semantically similar clinical notes. However, larger models require substantially greater computational resources and longer inference times, whereas smaller models generally require fewer computational resources and may be faster and more efficient to deploy. Iterating on prompts with smaller models allows rapid experimentation and more efficient debugging of prompt structure, classification criteria, and output formatting.

#### Practical Model Comparison Strategy

When evaluating alternative models, comparisons should be conducted under controlled conditions. To ensure that performance differences reflect model capabilities rather than experimental variability, inference settings such as temperature, maximum token limits, and decoding parameters should remain consistent across models. The same stabilized prompt and final note dataset should also be used for evaluation. Model performance can then be compared using the same evaluation metrics used throughout the workflow, including accuracy, precision, recall, *F*_1_-score, and confusion matrices. In addition to classification performance, investigators may also track structural compliance by measuring JSON validity rates, schema violations, and parsing failures. Prediction stability can also be assessed by running repeated inference under deterministic decoding settings and measuring the consistency of model outputs. Maintaining consistent evaluation conditions ensures that observed differences in performance are attributable to the models themselves rather than changes in prompts, parameters, or datasets.

#### Selecting Candidate Models

Candidate models should be selected based on factors such as model size, instruction-tuning quality, context window length, and reliability of structured output generation. Public benchmarks (eg, massive multitask language understanding [MMLU], beyond the imitation game benchmark (BIG-Bench), and holistic evaluation of language models [HELM]) and model reports may provide general insight into reasoning and instruction-following behavior but are not validated proxies for performance specifically on psychiatric or behavioral health extraction tasks, which depend heavily on a model’s handling of negation, hedged language, and risk-related terminology rather than on general reasoning ability. Model selection should therefore be guided by task-specific evaluation on representative clinical datasets under controlled conditions.

Beyond model size, architecture and instruction-tuning differences affect structured output reliability and instruction-following behavior [17,18]. Some models process structured inputs, such as labeled prompt sections or JSON-formatted contextual information, more reliably than free-form text. Because these characteristics vary across model families and versions, model selection should be treated as an empirical decision and evaluated under controlled conditions using identical prompts and prompt development sets.

### Example

Prompt development for both tasks used a zero-shot strategy: predictions were guided entirely by natural-language instructions, with no labeled examples and no task-specific fine-tuning. Starting from the baseline prompts (see Create a Baseline Prompt), refinement addressed classification behavior, output structure, and model selection.

Classification refinement targeted early errors involving negated statements (eg, documentation denying suicidal ideation) and historical symptom references by adding explicit inclusion and exclusion criteria. The final SITB prompt specifies “Classify as No only if the patient denies suicidal ideation and no self-injury behavior is described,” and the adherence prompt classifies a case as nonadherent when “missed doses, skipped doses, or inconsistent medication use” are documented. Stability was verified by running repeated inference on the prompt development set under deterministic decoding; for the pediatric SITB task, the confusion matrix (50 true positives, 45 true negatives, 5 false positives, and no false negatives) was unchanged across repeated runs.

Prompts were also revised to require strict JSON output, replacing the free-form, explanation-embedded responses seen during early development (eg, “The patient denies suicidal ideation, so the answer is No”): {“likert_score”: −3|...|3, “likert_score_analysis”: “<one short sentence>“} for Likert mode, {“label”: “Yes”/“No”} for binary mode. Prompts were further organized into clearly labeled, delimited sections (eg, a “Clinical Note”: section with quotation-enclosed text) to separate instructions from source content and reduce parsing ambiguity across batches. For Likert mode, a binary decision threshold (τ) was selected on the prompt-development set by maximizing the Youden J statistic (sensitivity+specificity−1) across candidate thresholds s ∈ {−3,...,3} [19], fixed before evaluation and not adjusted afterward. Refinement continued iteratively until classification performance, stability, and structural compliance stabilized on the prompt development set; the frozen prompts were then applied unchanged to the evaluation datasets. Full prompt text for both tasks, including the best-practice template comparison, is provided in Multimedia Appendix 3.

For model selection, we chose Llama 3.2 and Llama 3.3 over domain-adapted clinical models (eg, BioMistral, ClinicalBERT variants). Both Llama models are available via lightweight local inference frameworks (eg, *Ollama*), can be deployed in quantized form on consumer and institutional hardware, and demonstrate strong zero-shot instruction-following behavior [20]. Domain-adapted clinical models, by contrast, are less broadly accessible and are not consistently maintained across institutions. Teams with access to such models may find that domain adaptation improves performance on tasks involving specialized terminology; this remains an open question for future evaluation. Smaller instruction-tuned models (eg, Phi-3 and Mistral 7B) are viable alternatives for more constrained hardware.

**Figure 4.**
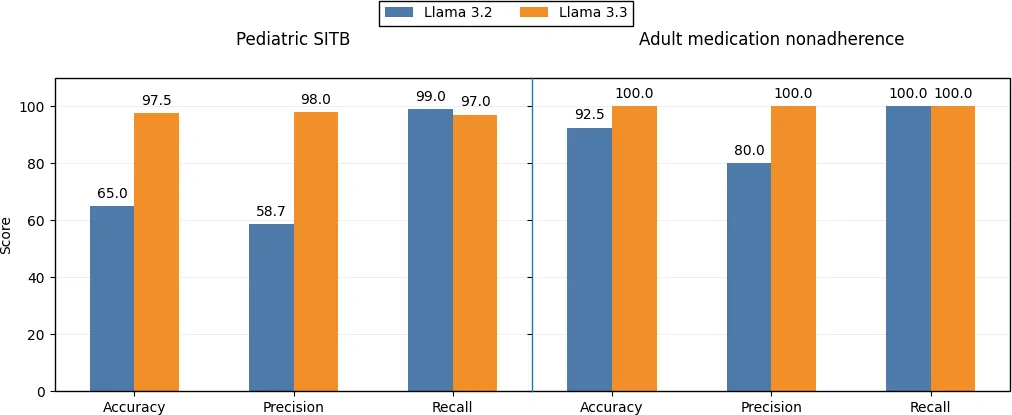
Comparison of zero-shot binary classification performance (accuracy, precision, and recall) for Llama 3.2 and Llama 3.3 across the pediatric self-injurious thoughts and behaviors (SITB) and adult medication nonadherence datasets. SITB: self-injurious thoughts and behaviors.

Llama 3.2 supported rapid iterative refinement during development. Once prompts and schemas were frozen, they were evaluated unchanged on Llama 3.3 under identical conditions so that any performance difference could be attributed to model capability rather than experimental variability. As shown in Figure 4, Llama 3.3 outperformed Llama 3.2 on both tasks. For pediatric SITB (Likert mode), accuracy rose from 65.0% to 97.5% and precision from 58.7% to 98.0%, while recall stayed stable (99.0% vs 97.0%). For antipsychotic nonadherence, accuracy rose from 92.5% to 100.0% and precision from 80.0% to 100.0%, while recall stayed unchanged at 100.0%.

## Results

### Overview

Once the prompts are frozen and applied unchanged to the test data, the classification performance is measured under controlled inference conditions. The goal is to determine whether the prompt design, output schema, and model selection produce reliable predictions for the target clinical task. In this workflow, the system flags notes for clinician review; it does not make autonomous clinical determinations.

### Evaluate Classification Performance

All evaluations were conducted on synthetic clinical notes under deterministic inference settings; performance estimates should be interpreted as controlled workflow-validation results rather than real-world deployment benchmarks (see Common Pitfalls and Limitations).

After prompt refinement and model selection were completed (Steps 1‐2), the finalized prompts were applied unchanged to the evaluation datasets using the Llama 3.3 model under deterministic inference settings.

Classification performance was evaluated using accuracy, precision, recall, and confusion matrices for the binary prompting condition. For Likert prompting, ordinal scores were converted to binary predictions using the Youden-optimal threshold determined during development. Likert scores were binarized using τ=−1, the Youden-optimal threshold selected on the prompt development set prior to evaluation (see Prompt Finalization).

Because the evaluation datasets consisted of synthetic notes generated under task-specific specification sheets, the following performance estimates reflect performance on controlled synthetic test sets and should not be interpreted as deployment benchmarks. Table 2 summarizes the overall classification accuracy for both tasks under binary and Likert prompting modes. Across both clinical domains, the structured prompting pipeline achieved high performance with minimal task-specific modification.

**Table 2.** Classification performance for pediatric SITB^a^ detection and adult medication nonadherence using Llama 3.3.

Output format	Accuracy (95% CI)^b^	Precision^c^ (95% CI)	Recall^c^ (95% CI)	*F*_1_-score^c^ (95% CI)
Pediatric SITB				
Likert^d^	0.975 (0.943-0.989)	0.980 (0.929-0.994)	0.970 (0.915-0.990)	0.975 (0.949-0.995)
Binary^e^	0.970 (0.936-0.986)	0.943 (0.881-0.974)	1.000 (0.963-1.000)	0.971 (0.947-0.991)
Adult medication nonadherence				
Likert	1.000 (0.980-1.000)	1.000 (0.940-1.000)	1.000 (0.940-1.000)	1.000 (1.000-1.000)
Binary	1.000 (0.980-1.000)	1.000 (0.940-1.000)	1.000 (0.940-1.000)	1.000 (1.000-1.000)

a SITB: self-injurious thoughts and behaviors.

b CIs reflect sampling uncertainty on 200 synthetic notes per task and do not represent estimates of real-world performance variability.

c Precision, Recall, and *F*_1_-score values of 1.000 indicate perfect classification on the test set.

d Likert: Likert-scale prompting configuration.

e Binary: binary prompting configuration.

**Figure 5.**
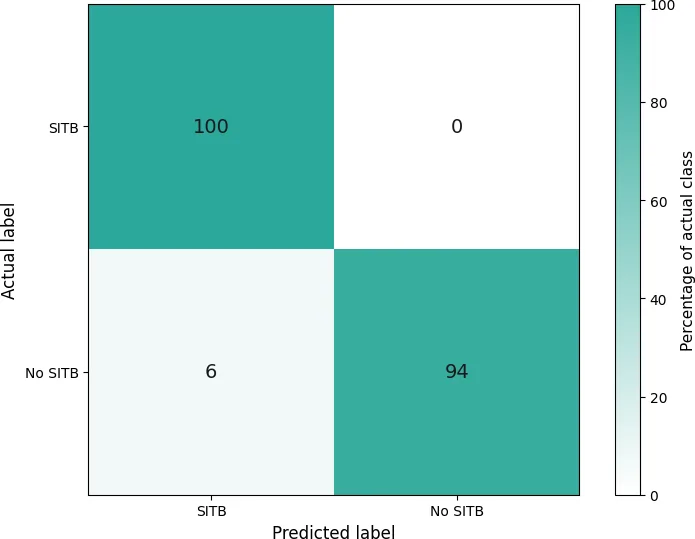
Confusion matrix for pediatric self-injurious thoughts and behaviors (SITB) classification. SITB: self-injurious thoughts and behaviors.

Table 2 reports accuracy, precision, recall, and *F*_1_-score for both prompting modes, with CIs reflecting sampling uncertainty on the 200-note evaluation set. The overall pattern is more important than any single number. As shown in the confusion matrix (Figure 5), the model produced zero false negatives and a small number of false positives (6/200), meaning every true SITB-positive case was flagged for review, at the cost of roughly 1 in 17 flagged notes not meeting SITB criteria. That reflects the intended trade-off of recall-prioritized tuning; for a safety-critical task, missing a positive case is treated as more costly than an occasional unnecessary review.

For the antipsychotic medication nonadherence task, the model achieved perfect classification under both prompting modes on this evaluation set (see Table 2). This result reflects the relative simplicity of distinguishing adherence from nonadherence language in notes generated according to specification and should not be treated as a clinical benchmark. Perfect classification on a controlled synthetic set is a common finding when the evaluation set does not capture the full ambiguity of real clinical documentation; validation on real clinical notes is necessary before these results can be interpreted as estimates of deployment performance.

**Figure 6.**
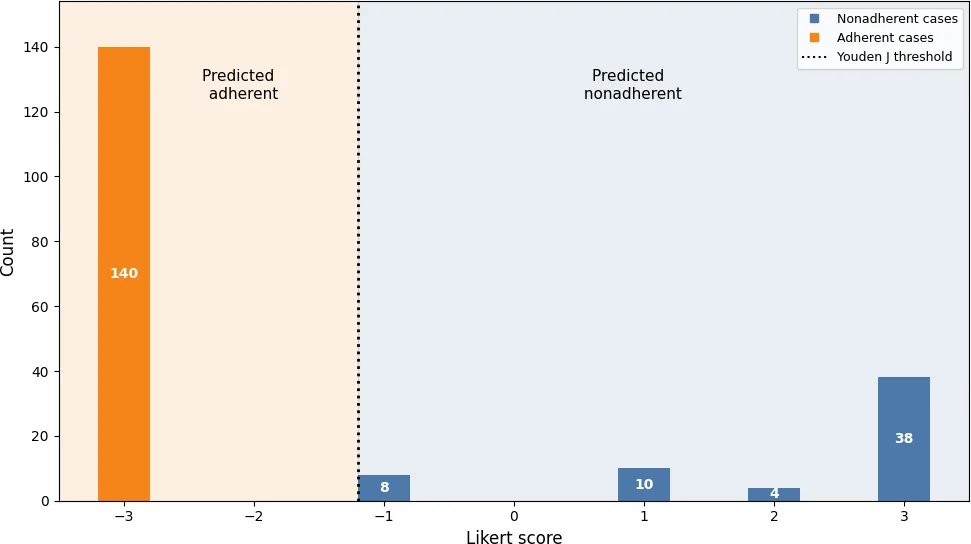
Distribution of ordinal Likert scores for the adult medication nonadherence task stratified by ground-truth label, with the Youden-optimal threshold indicated.

**Figure 7.**
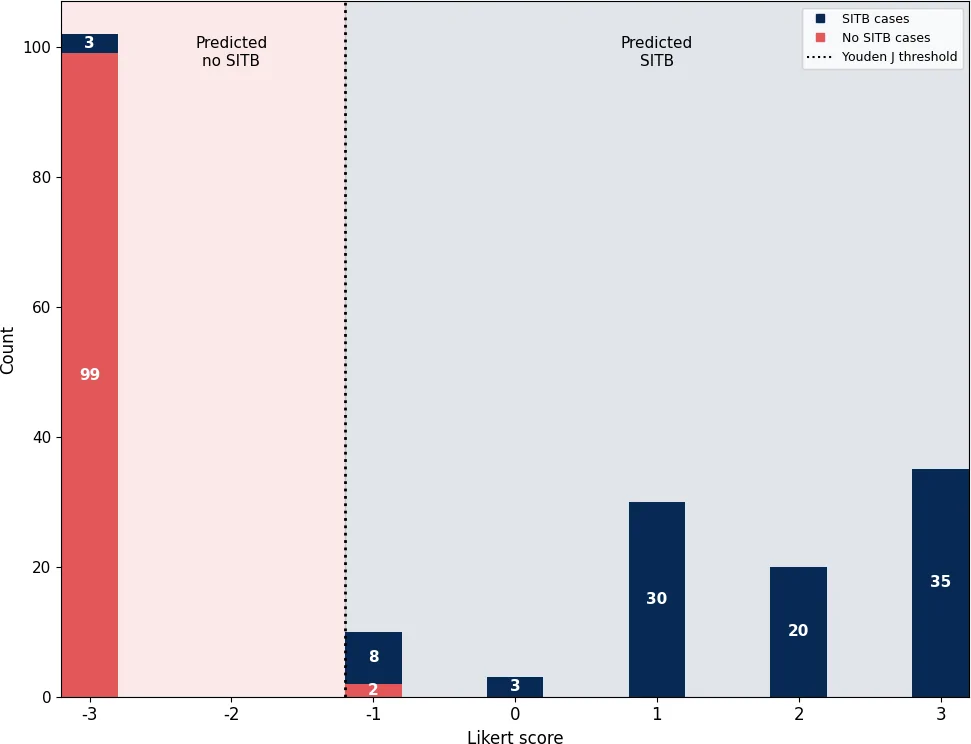
Distribution of ordinal Likert scores for the pediatric self-injurious thoughts and behaviors (SITB) task stratified by ground-truth label, with the Youden-optimal threshold indicated. SITB: self-injurious thought and behavior.

Figure 6 and Figure 7 illustrate the distribution of Likert scores assigned by the model for each task. In both datasets, the model assigned clearly differentiated scores to positive and negative cases. Negative examples were concentrated at the lowest Likert score (−3), indicating strong evidence against the target condition, while positive cases were predominantly assigned scores of 1‐3, indicating graded evidence in favor. The Youden-optimal threshold (τ=−1) fell between these clusters, allowing ordinal outputs to be converted into binary predictions without substantial loss of classification performance. This separation suggests the model’s confidence is well calibrated on synthetic notes.

These results indicate that the prompt-based workflow can produce consistent and well-separated classification behavior under controlled synthetic evaluation conditions using zero-shot prompting. Further validation on real clinical datasets is required to assess performance under real-world deployment conditions.

### Characterize Failure Modes

#### Overview

Qualitative error analysis examines cases where the model’s prediction disagrees with the reference labels. Examining these failure modes helps identify patterns in model reasoning, prompt interpretation, or dataset labeling that may influence performance. For safety-critical tasks, the specific types of errors may matter more than aggregate accuracy; for example, a pipeline that misses SITB creates a different risk from one that overflags a note for review. Error analysis should therefore characterize not only how many errors occurred but also what clinical scenarios produced them so that deployment teams can design appropriate human oversight.

To illustrate the error analysis process, we examined cases where model predictions disagreed with ground-truth labels under both the binary and Likert prompting schemes. A small number of misclassifications occurred in the pediatric SITB task. These errors were not randomly distributed but instead clustered in clinically nuanced cases where documentation patterns conflicted with the task labeling rules.

Under the ground-truth labeling scheme, a case was considered positive if the clinical note documented suicidal ideation, suicidal behavior, or nonsuicidal self-injury (NSSI), even when suicidal intent was explicitly denied. This labeling decision is consistent with the Centers for Disease Control and Prevention (CDC) Self-Directed Violence Surveillance definitions [21] and the Columbia Classification Algorithm [22], which treat NSSI and suicidal ideation as distinct but related constructs warranting identification. However, collapsing these clinically distinct categories (NSSI, suicidal ideation, and suicide attempts) into a single positive class necessarily obscures the specific behavior the model is detecting; future work may benefit from reporting performance separately by SITB subtype.

#### False Negatives

Two of the misclassified cases involved patients engaging in self-injurious behaviors used for emotional regulation while simultaneously denying suicidal intent. In one case, the patient repeatedly burned their forearm with a lighter during episodes of emotional distress, explaining that the behavior served as a coping mechanism rather than an attempt to die (case: child 114). In another case, the patient reported scratching her arms with a paperclip until bleeding in order to “release pressure” during periods of academic and social stress (case: child 130). Although both behaviors met the study’s criteria for NSSI and were therefore labeled as positive SITB cases, the model occasionally classified these notes as negative. We hypothesize that the model weighted explicit denial statements (eg, “not trying to kill myself”) more strongly than the documented behavioral evidence of self-injury, despite the prompt instructions specifying that behavioral evidence should override denial of suicidal intent.

Another misclassification involved passive death-related language embedded within a clinically documented grief reaction. In this case, the patient expressed wishes to be reunited with a recently deceased companion animal and reported thoughts about wanting to die in order to be with the pet again, while explicitly denying suicidal intent (case: child 82). This case is mechanistically distinct from the NSSI errors. Although passive death wishes meet the task criteria for a positive label, the surrounding clinical context, particularly documentation emphasizing normative grief and the absence of suicidal planning, appears to have influenced the model toward a negative classification. This suggests that contextual framing within clinical narratives can attenuate suicide-related signals during model reasoning, even when the language itself meets predefined detection criteria.

#### False Positives

The 6 false positive notes involved cases where emotional distress language was present without documented SITB, reflecting a tendency to overidentify nonspecific affective distress as risk-relevant content. This corresponds to a false positive rate of approximately 6% on the synthetic evaluation set. In a deployment context, this would translate to roughly 1 in 17 flagged notes requiring clinician review without meeting criteria, representing an expected trade-off of sensitivity-favoring behavior. Such overidentification has direct implications for triage workload and should be considered when calibrating decision thresholds. Detailed case-level logging was not retained in this tutorial evaluation; future implementations should preserve predictions and source text to enable systematic audit and refinement.

#### Model Version Effects

In the final evaluation, Llama 3.3 achieved perfect classification on the antipsychotic medication nonadherence task. However, error analysis during development with the earlier Llama 3.2 model revealed a systematic failure pattern worth highlighting as a tutorial lesson. Llama 3.2 misclassified several notes describing acute psychiatric decompensation despite explicitly documented adherence. These cases frequently included strong adherence verification (eg, caregiver-supervised dosing, directly observed administration, and/or therapeutic antipsychotic drug levels) while simultaneously describing severe symptoms, hospitalization planning, or safety interventions. The co-occurrence of clinical deterioration with confirmed adherence appeared to create conflicting signals that the smaller model resolved incorrectly. Llama 3.3, with its larger parameter count, corrected this. The finding illustrates a practical lesson; model version changes (even within the same model family) can silently alter clinically meaningful behavior. Re-evaluation of known failure-mode cases is a prudent step for teams adapting workflows across different models or model versions.

#### Summary of Failure Patterns

Taken together, these qualitative observations suggest several plausible mechanisms for misclassification across the 2 tasks. In the pediatric SITB task, errors arose when (1) explicit denial statements were prioritized over behavioral evidence of self-injury or (2) contextual framing attenuated the salience of death-related language that met the labeling criteria. In the antipsychotic medication nonadherence task, early errors occurred when severe psychiatric deterioration co-occurred with documented adherence, creating conflicting signals. Because only a small number of errors were observed overall, these interpretations should be considered hypotheses rather than definitive failure patterns. Nevertheless, the concentration of errors within these specific clinical scenarios highlights areas where prompt instructions, classification criteria, or human-in-the-loop safeguards should be strengthened before any deployment consideration. It is also important to note that ground-truth labels on synthetic notes reflect the specification sheet rules used during generation rather than independent clinical consensus. Disagreement between model and label may reflect labeling ambiguity rather than purely model error.

## Discussion

### Principal Findings

Pediatric and adult behavioral health documentation differ substantially in structure, tone, and clinical cues, yet applying the same pipeline to both tasks demonstrates that a schema-driven approach grounded in strict JSON outputs and robust parsing can support multiple clinical inference tasks with minimal modification beyond redefining schemas and label policies. This reusability has its limits; the pipeline as described is for single-construct binary or ordinal classification. Tasks requiring temporal reasoning across multiple notes, quantitative extraction (eg, medication dosing), or multilabel phenotyping would necessitate architectural modifications not covered here.

From an implementation perspective, 3 design decisions did most of the work in making this pipeline reliable. First, schema-driven task definition anchored the clinical construct, output format, and evaluation to a single specification, keeping labels, prompts, and metrics aligned with the same objective. Second, strict JSON-only output constraints, with automated extraction of the first valid JSON object, made large-scale reproducible evaluation possible when free-text outputs would not. Third, local deployment provided a privacy-preserving pattern aligned with how health systems would operationalize similar workflows on real clinical data, though in practice it requires on-premises or private-cloud GPU resources, institutional IT and security review, and verification that model licensing terms permit clinical use.

In applied settings, outputs from zero-shot LLM pipelines should be treated as decision-support signals rather than definitive clinical determinations [23]. A positive flag from this pipeline should trigger clinician review of the source note, not an automated action on the patient. No output should serve as the sole or primary basis for clinical decisions until prospective validation on real annotated notes has occurred. Practical deployment should incorporate guardrails such as (1) thresholding ordinal scores to prioritize sensitivity for safety-critical tasks, (2) human-in-the-loop workflows with explicitly defined roles and response protocols (eg, designating an individual as the reviewer, specifying a maximum review time window, defining the communication channel, and requiring documentation of the clinician’s response), (3) audit log requirements, including, at minimum, note IDs, model version, prompt version, timestamps, predictions, and applied thresholds, as well as the note text segment that triggered the extraction and prompt version change-control documentation, and (4) reference to existing clinical decision-support frameworks [24]. Further, the system should implement a fail-safe mode such that if schema validation fails or model confidence falls below the operating threshold, the output should surface as indicative of failed extraction rather than returning a null or default value that a clinician may act upon.

### Common Pitfalls and Limitations

All performance metrics reported in this tutorial were obtained from synthetically generated clinical notes with labels assigned by construction and should be interpreted as controlled workflow-validation results rather than estimates of clinical deployment performance. Although synthetic evaluation is useful for testing schema design, prompting logic, parsing, and error-handling under reproducible conditions, it cannot fully reproduce the linguistic variability, ambiguity, documentation noise, prevalence structure, and institutional heterogeneity of real clinical records, a gap that matters more for behavioral health documentation than for many other note types, given its reliance on narrative, indirect, and negation-heavy language to convey risk-relevant information.

The synthetic notes for the medication nonadherence task specified a single antipsychotic per patient, whereas individuals with serious mental illness are frequently prescribed multiple medications, including more than one antipsychotic. We acknowledge this simplification, which was made to keep the tutorial’s worked example easy to follow rather than to reflect the full complexity of real notes. The underlying workflow does not depend on this simplification: the same process of drafting an initial prompt, refining it against a prompt development set, and then evaluating on a final evaluation dataset applies regardless of how many medications or antipsychotics appear in a note, with the schema and label logic being adjusted accordingly. The reported performance metrics, however, may well depend on it. With a single named antipsychotic per note, the model is never required to determine which agent an adherence statement refers to. In real documentation, adherence may be described generically (“has not been taking his medications”), attached to one agent while others are continued, or implied only indirectly through symptom descriptions, refill gaps, or missed long-acting injectable appointments. Resolving which drug a statement implicates and whether nonadherence to one agent constitutes nonadherence to the antipsychotic regimen is a substantially harder inference than the one evaluated here. Accuracy, precision, and recall may differ in that setting, and a multimedication application would likely require additional pipeline components, such as resolving the medication list prior to classification, which we do not evaluate here.

In practice, several additional pitfalls may arise when applying this approach to real clinical data. First, performance may degrade when encountering out-of-distribution language, including institution-specific abbreviations or documentation styles not reflected in the synthetic data. Second, class prevalence in real-world settings is often highly imbalanced, which can substantially affect precision-recall trade-offs and requires threshold calibration tailored to the deployment context. Third, LLM outputs may exhibit variability across runs or models, necessitating normalization and validation layers to ensure consistent structured outputs. Finally, prompt sensitivity and schema design choices can meaningfully impact extraction performance, highlighting the need for iterative testing and evaluation on representative datasets. For examples of application to real clinical notes, see [25,26].

Aggregate metrics do not capture differential performance across patient subgroups. LLM performance on clinical notes may vary by patient age, race, ethnicity, primary language, provider type, and documentation style, and aggregate metrics may mask disparities. Documentation culture and note type matter as much as demographics; for example, an ED note, a crisis unit note, and an outpatient therapy note may describe the same clinical presentation in structurally different language, and a prompt validated on one note type may underperform on others. Before deployment, teams should stratify evaluation metrics by relevant subgroups (eg, age, primary language, note author role, and clinical setting) and treat acceptable subgroup performance as a prerequisite for deployment, rather than a post hoc audit.

Zero-shot inference can overinterpret ambiguous language, and intermediate Likert scores in particular should be interpreted cautiously [26,27]. In the pediatric SITB cases, misclassifications often arose from subtle or indirect expressions of distress, such as passive death wishes or ambiguous emotional statements [22] that did not explicitly indicate intent or behavior. Similarly, in antipsychotic nonadherence documentation, phrases such as “missed a few doses,” “ran out of refills,” or incomplete medication histories frequently led to intermediate Likert scores, reflecting genuine uncertainty in the narrative. Teams should consider including an “indeterminate” category in the output schema to trigger human review rather than forcing the model to commit to a classification it cannot support.

This is compounded by a critical scope boundary. No clinician ratings were collected to validate these intermediate Likert judgments, and until a validated mapping between the model’s ordinal outputs and established clinical instruments is established through independent clinical rating, Likert scores from the pipeline should not be surfaced to clinicians as severity ratings or used to inform triage or level-of-care decisions. Real-world use requires clinician validation, including mapping to established clinical vocabularies with ordinal categories (eg, Columbia Suicide Severity Rating Scale).

### Practical Guidance and Next Steps

Ensuring programmatic schema validation should be considered mandatory for workflows relying on structured outputs, as strict format compliance enables reliable parsing and evaluation at scale. Local deployment of inference models similarly supports PHI-safe workflows and aligns with institutional auditing and compliance requirements. Any change to a prompt in a deployed system should be treated as a functionally significant system update, not a low-stakes configuration change.

Once reliable extraction of signals such as SITB or antipsychotic medication nonadherence becomes feasible, multiple downstream applications become possible. These applications carry different levels of patient safety risk and must be considered in a staged manner. Lower-risk applications (eg, retrospective quality improvement) are appropriate starting points. Higher-risk applications (eg, real-time triage flagging) should be undertaken only after prospective validation with subgroup-stratified performance and formal institutional governance approvals in place.

We suggest the minimum viable local validation should include (1) a deidentified sample of at least 100 local clinical notes representative of the target documentation type; (2) independent annotation by 2 qualified clinicians using a structured rubric, with interrater reliability reported (κ ≥0.70 recommended); (3) prespecified performance thresholds (eg, sensitivity ≥0.85 for safety-critical SITB flagging) established before evaluation, not after; (4) stratified reporting of performance by relevant patient subgroups; (5) documentation of failure cases and their clinical characteristics; and (6) a defined revalidation trigger when the model version, prompt text, or target note population changes.

### Conclusion

We present a PHI-safe, reproducible workflow for extracting clinically relevant information from behavioral health notes using open-source LLMs deployed within institutional computing environments. Across 2 clinical examples, the tutorial shows that schema-driven prompting, structured outputs, and automated validation can support controlled end-to-end workflow testing with minimal task-specific modification. Because evaluation was conducted on synthetic notes with labels assigned by constructions, the reported performance should be interpreted as proof of workflow feasibility under controlled conditions, not as evidence of real-world clinical accuracy. No clinical benefit, efficiency gain, or safety improvement can be claimed without prospective validation on real-world clinical notes. Future work should validate the same pipeline on clinician-annotated real clinical notes across institutions and documentation settings.

Rather than replacing clinical judgment, such workflows can provide scalable decision-support signals that enable safer triage, monitoring, and quality improvement efforts. We provided the synthetic clinical notes generated and used during this study in the Zenodo (European Organization for Nuclear Research [CERN]) repository [28]. By emphasizing structured outputs, automated validation, and local deployment, this tutorial aims to provide a practical foundation that clinicians and health systems can adapt to future clinical NLP tasks as LLM capabilities continue to evolve.

## Supplementary material

10.2196/97498 Multimedia Appendix 1 Detailed procedures for gathering real clinical notes or generating labeled synthetic notes for the 2 illustrative tasks.

10.2196/97498 Multimedia Appendix 2 Specification sheet for synthetic psychiatric progress note generation: antipsychotic medication nonadherence detection task.

10.2196/97498 Multimedia Appendix 3 Prompt Templates: Best-Practice Structure and Task-Specific Implementations.

## Abbreviations

BERT: Bidirectional Encoder Representations from Transformers
BIG-Bench: beyond the imitation game benchmark
BioClinicalBERT: Biomedical and Clinical Bidirectional Encoder Representations from Transformers
CDC: Centers for Disease Control and Prevention
CERN: European Organization for Nuclear Research
CFR: Code of Federal Regulations
ClinicalBERT: Clinical Bidirectional Encoder Representations from Transformers
CPU: central processing unit
ED: emergency department
GPU: graphics processing unit
HELM: holistic evaluation of language models
HIPAA: Health Insurance Portability and Accountability Act
LLM: large language model
MMLU: massive multitask language understanding
NLP: natural language processing
NSSI: nonsuicidal self-injury
PHI: protected health information
SITB: self-injurious thoughts and behaviors

## References

[1] Patra BG, Lepow LA, Kasi Reddy Jagadeesh Kumar P (2025). Extracting social support and social isolation information from clinical psychiatry notes: comparing a rule-based natural language processing system and a large language model. J Am Med Inform Assoc.

[2] Vance LA, Way L, Kulkarni D (2025). Natural language processing to identify suicidal ideation and anhedonia in major depressive disorder. BMC Med Inform Decis Mak.

[3] Kariotis TC, Prictor M, Chang S, Gray K (2022). Impact of electronic health records on information practices in mental health contexts: scoping review. J Med Internet Res.

[4] Locke S, Bashall A, Al-Adely S, Moore J, Wilson A, Kitchen GB (2021). Natural language processing in medicine: a review. Trends Anaesth Crit Care.

[5] Jin Y, Liu J, Li P (2025). The applications of large language models in mental health: scoping review. J Med Internet Res.

[6] McCoy TH, Perlis RH (2025). Applying large language models to stratify suicide risk using narrative clinical notes. J Mood Anxiety Disord.

[7] Holmes G, Tang B, Gupta S, Venkatesh S, Christensen H, Whitton A (2025). Applications of large language models in the field of suicide prevention: scoping review. J Med Internet Res.

[8] Wiest IC, Verhees FG, Ferber D (2024). Detection of suicidality from medical text using privacy-preserving large language models. Br J Psychiatry.

[9] Goel A, Gueta A, Gilon O (2023). LLMs accelerate annotation for medical information extraction. Proceedings of Machine Learning Research.

[10] Wiest IC, Wolf F, Leßmann ME (2025). A software pipeline for medical information extraction with large language models, open source and suitable for oncology. NPJ Precis Oncol.

[11] Adhikary PK, Srivastava A, Kumar S (2024). Exploring the efficacy of large language models in summarizing mental health counseling sessions: benchmark study. JMIR Ment Health.

[12] Liu L, Lian L, Hao Y (2025). Human level information extraction from clinical reports with finetuned language models. Sci Rep.

[13] Liu P, Yuan W, Fu J, Jiang Z, Hayashi H, Neubig G (2021). Pre-train, prompt, and predict: a systematic survey of prompting methods in natural language processing. arXiv.

[14] Brown TB, Mann B, Ryder N (2020). Language models are few-shot learners.

[15] Wei J, Wang X, Schuurmans D (2022). Chain-of-thought prompting elicits reasoning in large language models. http://www.proceedings.com/68431.html.

[16] Wei J, Tay Y, Bommasani R (2022). Emergent abilities of large language models. Trans Mach Learn Res.

[17] Ouyang L, Wu J, Jiang X (2022). Training language models to follow instructions with human feedback. http://www.proceedings.com/68431.html.

[18] Geng S, Cooper H, Moskal M (2025). JSONSchemaBench: a rigorous benchmark of structured outputs for language models. arXiv.

[19] Fluss R, Faraggi D, Reiser B (2005). Estimation of the Youden Index and its associated cutoff point. Biom J.

[20] Grattafiori A, Dubey A, Jauhri A (2024). The Llama 3 herd of models. arXiv.

[21] Crosby AE, Ortega L, Melanson C (2011). Self-directed violence surveillance: uniform definitions and recommended data elements, Version 1.0. Centers for Disease Control and Prevention; 2011. CDC Stacks.

[22] Posner K, Oquendo MA, Gould M, Stanley B, Davies M (2007). Columbia Classification Algorithm of Suicide Assessment (C-CASA): classification of suicidal events in the FDA’s pediatric suicidal risk analysis of antidepressants. Am J Psychiatry.

[23] Edgcomb JB, Saha A, Klomhaus A (2026). Detecting pediatric emergency service use for suicide and self-harm: multimodal analysis of 3828 encounters. JMIR Ment Health.

[24] Reddy S, Allan S, Coghlan S, Cooper P (2020). A governance model for the application of AI in health care. J Am Med Inform Assoc.

[25] Patil A, Tao S, Gedhu A (2025). Evaluating reasoning LLMs for suicide screening with the Columbia-Suicide Severity Rating Scale. arXiv.

[26] Thomas J, Elyoseph Z, Kuchinke L, Meinlschmidt G (2025). Large language model performance versus human expert ratings in automated suicide risk assessment. Sci Rep.

[27] Tam TYC, Sivarajkumar S, Kapoor S (2024). A framework for human evaluation of large language models in healthcare derived from literature review. NPJ Digit Med.

[28] Saha D, Edgcomb JB (2026). DiyadotSaha/Synthetic-Data: synthetic behavioral health clinical notes: SITB and medication adherence detection. Version 1.0.1.

