# Supplementary material for: Practical Guide to Large Language Models for Information Extraction in Behavioral Health Notes: Tutorial
Source: JMIR Ment Health. 2026 Sep 24;13:e97498. doi: 10.2196/97498 (PMC13614219; doi:10.2196/97498)
Supplement: Supplementary file 1 [file mental-v13-e97498-s002.pdf]

Synthetic Psychiatric Progress Notes Generation Specification

1. Purpose

Generate **fully synthetic**, **realistic**, **U.S.-style psychiatric outpatient progress notes** that mimic authentic clinical documentation for patients with **chronic psychotic disorders**. The notes must be **internally consistent** (all fields agree with each other), contain **rich narrative detail**, and follow the structure and style of real U.S. psychiatric documentation. All data must be **synthetic** — no real patient data.

---

2. Core Requirements

2.1 Output Format

- **One JSON file per patient** (ID001.json ▯ ID050.json)
- UTF-8 encoding
- Consistent structure matching the example provided
- Keys must exactly match the provided example (e.g., "Patient_Name", "Mental_Status_Examination")

2.2 Document Structure

Each file should contain:

```
{
 "document_type": "UCLA Psychiatry Outpatient Progress Note",
 "fields": {
  "Patient_Name": "First Last",
  "Patient_MRN": "ID###",
  "Date_of_Birth": "YYYY-MM-DD",
  "Date_of_Service": "YYYY-MM-DD",
  "Identifying_Data_Chief_Complaint": "...",
  "Collateral_Contact_Information": "...",
  "Subjective": "...",
  "Mental_Status_Examination": {
   "Appearance": "...",
   "Speech": "...",
   "Affect": "...",
   "Mood": "...",
   "Thought_Content": "...",
   "Thought_Process": "..."
```

```
    },
    "Behavioral_Health_Checkup": {
      "PHQ_9": int,
      "GAD_7": int,
      "DAST_10": int,
      "AUDIT_C": int,
      "C_SSRS": "..."
    },
    "Current_Medications": "...",
    "CURES_Report": "...",
    "Laboratory_Data": {
      "Recent_Lab_Results": "...",
      "Recent_Imaging_Results": "..."
    },
    "Diagnostic_Impression": "...",
    "Psychosocial_Context": "...",
    "Assessment_and_Plan": [...],
    "RTC": "...",
    "Author": "Note written by <Provider_Name>, <Role>",
  }
}
```

---

3. Content Generation Rules

3.1 Patient Demographics

- **Age:** ≥ 18 years old
- Weighted distribution:
  - **25–60 years**: majority (~70%)
  - **>60 years**: minority (~20%)
  - **18–24 years**: minority (~10%)

- **DOB & Age must match**
- **Names:** Realistic, U.S.-style, ethnically diverse (e.g., Anglo-American, African-American, Hispanic, Asian-American, etc.)
- **Gender:** ~50% male / 50% female
- **Emergency Contact:**
  - Format: "Emergency Contact: <Relation> (<Full Name>) at 555-XXX-XXXX"
  - 555 area code for safety
- **Provider Name & Role:**
  - Random realistic U.S.-style provider names
  - Roles: Psychiatrist, Nurse Practitioner, Pharmacist, Care Coordinator

---

3.2 Diagnosis

- Always
  one
  qualifying chronic psychotic disorder:
  - "Schizophrenia"
  - "Schizoaffective Disorder"
  - "Other chronic psychotic disorder"

3.3 Medications

- Always **one antipsychotic** from antipsychotic_medications.csv
- Medication name in Current_Medications
- Consistent with narrative in **Subjective** and **Assessment & Plan**

3.4 Adherence

- **30%**
  of patients =
  non-adherent

  - Randomly select from: "Cost", "Side effects", "Complexity", "Forgetfulness"
  - Must appear in both:
    - **Subjective**: "Patient reports missing doses because..."
    - **Assessment & Plan**: should reference non-adherence if present, but not repeat explicit subjective statements.

  - Medication dose details should only appear in the Subjective section. Do not mention specific dose information in other sections.
  - Each note should feature unique patient information and not reuse names or content across files.

3.5 Narrative Sections

Subjective

- Rich, detailed narrative of 100–200 words. In Subjective, do not use explicit statements like "I have not been adhering to my medication." Instead, patients may describe occasionally missing or forgetting doses, but should not directly state that they are non-adherent.
- Includes:
  - Interview context
  - Symptom change since last visit
  - Medication dose details may only appear here
  - Medication adherence (do not include explicit statements of non-adherence; patients may mention missing or forgetting doses, but not state they are generally non-adherent)
  - Sleep pattern & changes
  - Hallucinations / paranoia
  - Mood rating
  - Functioning/ADLs
  - Substance use status
  - Paranoid/delusional ideation if relevant
  - Risk assessment
  - Appetite/weight

Mental Status Examination

- Populate dropdown placeholders with **one valid option** from each {} set
- Replace *** with realistic clinician-entered text

Behavioral Health Checkup

- Realistic
  numeric scores
  :
  - PHQ-9:
    - Stable patients: 0–5
    - Depressed patients: 10–27

  - GAD-7:
    - Stable: 0–5
    - Anxious: 8–21

  - DAST-10: usually 0 unless substance use mentioned
  - AUDIT-C: 0–12, skew low for stable patients
  - C-SSRS: "No suicidal ideation reported" unless risk noted

---

3.6 Laboratory & Imaging

- Usually empty or short placeholder text:

  - "Normal CBC. Elevated glucose."
  - "No acute findings on head CT"

3.7 Assessment & Plan

- Bullet-point list
- Always mirrors key findings from Subjective
- If non-adherence present ▯ explicitly addressed
- Include safety counseling and follow-up plan

---

4. Data Handling & Architecture

4.1 Data Sources

- antipsychotic_medications.csv ▯ select exactly 1 per patient
- Predefined lists for:
  - First names / last names (diverse)
  - Emergency contact relationships
  - Provider roles

4.2 Randomization

- Use weighted random generation for age distribution
- Randomly assign:
  - Diagnosis
  - Provider role
  - Adherence status & reason
  - Symptom severity
  - Visit date (spread over last few years)

4.3 Internal Consistency

- All fields must be internally consistent. For example, the medication in Current_Medications must match what is described in the Subjective and Assessment & Plan sections; demographic details, age, and date of birth must align; gender should match with name; and all narratives should be logically coherent with structured data.
- Age ↔ DOB consistency

- Gender ↔ name alignment
- Diagnosis ↔ symptoms ↔ behavioral scores alignment
- Adherence ↔ narrative ↔ plan alignment
- Date of service always after DOB & plausible

---

5. Error Handling

- Validate:
  - DOB yields correct age
  - Only one medication per patient
  - JSON structure matches schema

- Retry generation if:
  - Age < 18
  - Missing required field
  - Contradictory statements

---

6. Testing Plan

1. **Schema Validation** – check that all JSON files match expected keys/structure
2. **Internal Consistency Checks**
   :
   - DOB ▯ Age
   - Gender ▯ Name
   - Medication ▯ Narrative

3. **Content Review**
   :
   - Random sample review for realism
   - Check that dropdown values replaced correctly

4. **Statistical Checks**
   :
   - 30% non-adherence
   - 50/50 gender split
   - Age distribution matches specification

---

7. Deliverables

- ID001.json … ID0100.json
- All files zipped as synthetic_psych_notes.zip
- All fields populated according to specification
