# Supplementary material for: Practical Guide to Large Language Models for Information Extraction in Behavioral Health Notes: Tutorial
Source: JMIR Ment Health. 2026 Sep 24;13:e97498. doi: 10.2196/97498 (PMC13614219; doi:10.2196/97498)
Supplement: Supplementary file 2 [file mental-v13-e97498-s001.docx]

# Appendix 1. Extended Methods: Dataset Construction

This appendix provides extended methodological detail supporting the main manuscript, including the full procedure for gathering real clinical notes or generating synthetic ones with ground-truth labels.

Gather Notes

Prompt-based classification of clinical constructs requires a labeled dataset of notes. Each note must be paired with a ground-truth label that corresponds directly to the problem definition. This alignment ensures that the dataset, prompt instructions, and evaluation metrics remain structured around the same objective. Labels should reflect a clearly operationalized outcome that can be consistently evaluated rather than loosely associated clinical impressions. Such a dataset can be constructed in two primary ways: (1) extract and label real clinical documentation, or (2) generate synthetic clinical notes (when access, privacy, or outcome rarity constraints limit the use of real records).

### *Real Clinical Records*

In most applied settings, the starting point will be real clinical notes stored within an EHR system. Extraction should be conducted in collaboration with institutional data governance or informatics teams to ensure regulatory compliance.

Notes are typically retrieved through structured queries to a clinical data warehouse and exported in flat-file formats (e.g., comma-separated values (CSV) or JavaScript Object Notation (JSON)), with one row per note. Prior to LLM inference, all PHI should be removed or replaced using an approved de-identification pipeline. Each note should retain a unique encrypted identifier to enable linkage with ground-truth labels and downstream evaluation while preserving privacy. Any usage of real notes should occur under the auspices of institutional review board (IRB) approval or waiver, HIPAA compliance pathway, and data use agreements.

Ground-truth labels should be assigned through structured manual chart review by qualified clinicians or trained proxies, stored separately from note text, and documented with interrater reliability statistics (e.g., Cohen's Kappa). Where possible, definitions should align with validated constructs, for suicide-related behavior, the Centers for Disease Control and Prevention (CDC) Self-Directed Violence Surveillance Definitions [1] and the Columbia Classification Algorithm [2] provide established frameworks. Once de-identified, labeled, and formatted as a flat dataset, the notes are ready for prompt-based inference.

### *Synthetic Clinical Records*

In situations where real data are unavailable, limited, or constrained by privacy concerns, synthetic clinical notes may be generated. Synthetic datasets should approximate the structure, tone, and variability of real clinical documentation [3]. Specification sheets (“spec sheets”) serve as the structural foundation of synthetic note generation. These documents define required fields, semantic boundaries, narrative tone, and label-conditional constraints that specify what evidence must be present or absent for each class. By embedding label criteria directly into the generation process, ground-truth labels can be assigned at the time of data creation rather than inferred retrospectively. Because documentation style varies across clinical domains, task-specific spec sheets should be developed for each use case. An example specification sheet, for the antipsychotic medication nonadherence task, is provided in Appendix 3. Further information on generating high-quality notes and multi-faceted benchmarking of synthetic notes using LLMs can be found in recent literature [4,5].

### *Example*

To demonstrate the workflow described in this paper, we constructed synthetic clinical datasets for pediatric SITB detection (Task A) and antipsychotic medication nonadherence (Task B). We used two separate language models for generation to reduce the risk of generation–evaluation overlap. A GPT-family model (OpenAI; e.g., GPT-4-class model accessed via API) produced a preliminary prompt development set for iterative prompt refinement, while Claude Sonnet 4.5 (released Sept 2025) generated the final note corpus. This separation ensured that prompts were refined on data from a different model than the one used to generate the evaluation datasets, reducing the possibility that model-specific stylistic patterns would influence evaluation performance.

Each synthetic note was generated according to a structured specification sheet defining required metadata fields, narrative tone, and label-conditional constraints. The generation prompts contained three key components: (1) a strict JSON schema specifying the required fields and formatting for each note; (2) stylistic guidance derived from a single de-identified anchor note to approximate real clinical documentation style; and (3) task-specific constraints defining the evidence required for each label class. Generation instructions emphasized schema consistency while encouraging variability in narrative structure, wording, and phrasing across notes to minimize duplication and reduce the risk of overlapping or templated text patterns. We generated positive and negative cases in separate batches to minimize label contamination. Models were instructed to produce JSON-only outputs to allow automated schema validation. Notes failing validation were automatically discarded and regenerated. Each generated note included: (1) a synthetic identifier (serving as a proxy for patient ID); (2) the narrative clinical note text; and (3) a ground-truth classification label assigned by construction during generation. Ground-truth labels were stored separately from the note text to simulate real-world evaluation conditions. After validation, we aggregated notes into task-specific datasets, randomly shuffled them to eliminate ordering effects, and converted the structured fields into narrative note text before passing them to inference models for classification.

Class distributions were selected to balance controlled evaluation needs with clinically plausible prevalence. For the pediatric SITB task, we constructed a balanced dataset (50% positive, 50% negative) to approximate an enriched high-risk cohort consistent with emergency department screening populations, where rates of suicidal ideation and related behaviors are elevated [6]. This supports stable estimation of performance metrics, particularly recall, and clearer interpretation of classification behavior. For the medication nonadherence task, we used a moderately imbalanced distribution (approximately 30% nonadherent, 70% adherent), reflecting evidence that nonadherence affects a substantial minority of patients with serious mental illness [7]. Notes for this task were constructed to reference a single antipsychotic medication per patient; this simplification is discussed further in the Limitations section. Because class prevalence influences metrics such as precision and accuracy, balanced datasets support controlled evaluation, while imbalanced datasets better approximate deployment conditions [8].

For both tasks, we reserved 100 notes per task (50 positive, 50 negative) for prompt development and excluded these from all reported evaluation metrics. The development set was intentionally selected to include a range of documentation styles: clear positive cases, clear negative cases, borderline cases, and ambiguous documentation patterns. Examples included indirect SITB language in pediatric notes and partial adherence phrasing in antipsychotic nonadherence notes. We drafted initial prompts directly from the task definitions contained in the specification sheets using minimal instructional guidance in order to establish baseline model performance.

# References

1. Crosby AE, Ortega L, Melanson C. Self-Directed Violence Surveillance: Uniform Definitions and Recommended Data Elements, Version 1.0. Centers for Disease Control and Prevention, National Center for Injury Prevention and Control; 2011.<https://stacks.cdc.gov/view/cdc/11997>
2. Posner K, Oquendo MA, Gould M, Stanley B, Davies M. Columbia Classification Algorithm of Suicide Assessment (C-CASA): Classification of suicidal events in the FDA's pediatric suicidal risk analysis of antidepressants. Am J Psychiatry. 2007;164(7):1035-1043. doi:10.1176/ajp.2007.164.7.1035
3. Li J, Zhou Y, Jiang X, et al. Are synthetic clinical notes useful for real natural language processing tasks: A case study on clinical entity recognition. J Am Med Inform Assoc. 2021;28(10):2193-2201. doi:10.1093/jamia/ocab112
4. Yan C, Yan Y, Wan Z, et al. A multifaceted benchmarking of synthetic electronic health record generation models. Nat Commun. 2022;13(1):7609. doi:10.1038/s41467-022-35295-1
5. Yan C, Zhang Z, Nyemba S, Li Z. Generating synthetic electronic health record data using generative adversarial networks: Tutorial. JMIR AI. 2024;3:e52615. doi:10.2196/52615
6. King CA, Grupp-Phelan J, Brent D, et al; Pediatric Emergency Care Applied Research Network. Predicting 3-month risk for adolescent suicide attempts among pediatric emergency department patients. J Child Psychol Psychiatry. 2019;60(10):1055-1064. doi:10.1111/jcpp.13087
7. Higashi K, Medic G, Littlewood KJ, Diez T, Granström O, De Hert M. Medication adherence in schizophrenia: Factors influencing adherence and consequences of nonadherence, a systematic literature review. Ther Adv Psychopharmacol. 2013;3(4):200-218. doi:10.1177/2045125312474019
8. Saito T, Rehmsmeier M. The precision-recall plot is more informative than the ROC plot when evaluating binary classifiers on imbalanced datasets. PLoS One. 2015;10(3):e0118432. doi:10.1371/journal.pone.0118432
