# Supplementary material for: Practical Guide to Large Language Models for Information Extraction in Behavioral Health Notes: Tutorial
Source: JMIR Ment Health. 2026 Sep 24;13:e97498. doi: 10.2196/97498 (PMC13614219; doi:10.2196/97498)
Supplement: Supplementary file 3 [file mental-v13-e97498-s003.docx]

# Appendix 3: Prompt Templates

This appendix provides the final prompt templates used for the two illustrative classification tasks described in the main text: pediatric SITB detection (Table 1) and adult antipsychotic medication nonadherence detection (Table 2). Each table compares the implemented prompt against a best-practice template across system role, instructions, and output format.

**Table 1**

*Prompt structure for child SITB classification, comparing best practice template with implemented prompt by system role, instructions, and output format*

| **Best Practice Prompt** | **Prompt** |
| --- | --- |
| ***System Role***  You are a *<domain-specific role>*. Your task is to *<basic task>* | You are a psychiatric triage assistant. Your task is to determine whether the clinical note contains suicidal thoughts, suicidal ideation, suicide attempts, or intent to harm oneself whether current, past, passive, active, or historical. |
| ***Instructions (Binary mode)***  Classify as **Yes** if the input describes ANY of the following:  • *<target condition category 1>*  • *<target condition category 2>*  • *<related behaviors, subtypes, or indicators of the target condition>*    Classify as **No** only if ALL of the following are true:  • The input contains no evidence of  • The input contains no evidence of *<target condition category 1>*  • The input contains no evidence of *<target condition category 2>* | Classify as **Yes** if the note describes ANY of the following:  • Suicidal ideation (active or passive; current or recent), suicidal intent, planning, preparation, or a suicide attempt  • Self-harm / self-injury behavior (NSSI), even if described as coping or stress relief  • Self-injury urges with intent to harm oneself, even if suicidal intent is denied  • A clinical assessment/diagnosis explicitly documenting NSSI or self-harm    Classify as **No** only if:  • The note contains no suicidal ideation/intent/behavior AND no self-harm/NSSI behavior or urges  • The patient denies SI AND there is no self-injury behavior described |
| ***Instructions (Likert mode)***  Evaluate the provided *<input type>* for evidence of *<target condition>*. Assign a score using the following ordinal scale:  • *<positive scale values>*  • *<neutral value>*  • *<negative scale values>*    Additional requirements:  • If the input is ambiguous, use available contextual clues to assign the most appropriate score  • Base your decision solely on the information contained in the input  • Ignore any information unrelated to the target condition  • Consider both explicit statements and implicit contextual evidence  • Always assign exactly one score from the scale | Evaluate a medical record note for signs of Self-Injurious Thoughts or Behaviors (SITB). SITB includes:  • Suicide attempt: A potentially self-injurious behavior, associated with at least some intent to die, as a result of the act. A suicide attempt may or may not result in actual injury.  • Preparatory acts toward imminent suicidal behavior: The individual takes steps to injure him- or herself, but is stopped by self or others before harm has begun.  • Suicidal ideation: Passive thoughts about wanting to be dead or active thoughts about killing oneself, not accompanied by preparatory behavior.  • Non-suicidal self-injury (NSSI): Self-injurious behavior with no intent to die, intended to relieve distress or effect change in others.    Assign a score from the following Likert scale:  • 3 = Definitely SITB  • 2 = Very Probably SITB  • 1 = Probably SITB  • 0 = Possibly SITB  • −1 = Probably Not SITB  • −2 = Very Probably Not SITB  • −3 = Definitely Not SITB    Additional requirements:  • If the note is ambiguous, use contextual clues to assign your best estimate  • Ignore information unrelated to SITB; attend to any relevant abbreviations  • Protected health information has been replaced with placeholder tokens  • Always assign exactly one Likert score |
| ***Output Format***  **Mode = Likert:**  Return only JSON: {"likert_score": <integer in [given scale]>, "likert_score_analysis": <one short sentence>}    **Mode = Binary:**  Return only JSON: {"label": "<Yes or No>"}    *Do not include any extra text, explanations, markdown, or code fences.* | **Mode = Likert:**  Return only JSON with exactly these keys:  { "likert_score": −3 \| −2 \| −1 \| 0 \| 1 \| 2 \| 3,  "likert_score_analysis": <one short sentence> }    **Mode = Binary:**  Return only this JSON exactly:  {"label": "Yes"} or {"label": "No"}    *Do not include any extra text, explanations, markdown, or code fences.* |

**Note.** SITB = Self-Injurious Thoughts and Behaviors; NSSI = Non-Suicidal Self-Injury; SI = Suicidal Ideation. Italicized angle-bracket terms in the left column denote generalizable template placeholders. JSON output keys are shown verbatim as passed to the model.

**Table 2**

*Prompt structure for adult antipsychotic medical adherence classification, comparing best practice template with implemented prompt by system role, instructions, and output format*

| **Best Practice Prompt** | **Prompt** |
| --- | --- |
| ***System Role***  You are a *<domain-specific role>*. Your task is to *<basic task>* | You are a clinical decision-support assistant. Your task is to determine whether the patient described in the clinical note is adherent or non-adherent to their prescribed medications. Consider all documented behaviors, including missed doses, partial adherence, refill history, barriers to adherence (e.g., cost, access, side effects), and clinician or patient reports of medication-taking habits. |
| ***Instructions (Binary mode)***  • Classify as **Yes** (*<positive class label>*) if the input contains ANY of the following:  ◦ *<target condition category 1>*  ◦ *<target condition category 2>*  ◦ *<related behaviors, subtypes, or indicators>*  • Classify as **No** (*<negative class label>*) if the input contains ANY of the following:  ◦ The input contains no evidence of *<target condition category 1>*  ◦ The input contains no evidence of *<target condition category 2>*  Additional requirements:  • Base the classification only on the provided input  • Consider both explicit statements and implicit behavioral evidence  • Ignore information unrelated to the classification task  • Return exactly one classification label | Classify as **Yes** (Non-Adherent) if the note contains ANY of the following:  • Missed doses, skipped doses, or inconsistent medication use  • Running out of medication early or forgetting to refill prescriptions  • Taking medication less frequently than prescribed (e.g., "takes it only when needed")  • Stopping medication due to side effects, cost, or misunderstanding instructions  • Statements indicating poor routines, poor compliance, or inability to follow the plan  • Clinician impression explicitly describing non-adherence or poor compliance    Classify as **No** (Adherent) if the note contains ANY of the following:  • The patient reports taking medication as prescribed  • Consistent daily routine, timely refills, or documented adherence  • The clinician notes good compliance or stable adherence  • Minor delays (e.g., "took dose one hour late") that do NOT affect clinical adherence  • Ambiguous cases where medication use is mentioned but no signs of non-adherence appear    Important:  • If both adherent and non-adherent signals appear, classify according to the stronger evidence  • If uncertainty remains and any evidence of non-adherence is present, classify as Yes |
| ***Instructions (Likert mode)***  Evaluate the provided *<input type>* for evidence of *<target condition>*. Assign a score using the following ordinal scale:  *• <positive scale values>*  *• <neutral value>*  *• <negative scale values>*    Additional requirements:  • If the input is ambiguous, use available contextual clues to assign the most appropriate score  • Base your decision solely on the information contained in the input  • Ignore any information unrelated to the target condition  • Consider both explicit statements and implicit contextual evidence  • Always assign exactly one score from the scale | Evaluate a medical record note for signs of Medication Adherence Behavior (MAB). MAB key concepts include:  • Clear non-adherence: The patient intentionally or unintentionally fails to take medication as prescribed, including missed doses, inconsistent intake, early refills, running out of medication, stopping due to side effects, cost, misunderstanding, or poor routine.  • Partial or probable non-adherence: Patterns suggesting inconsistent adherence, but insufficient evidence for clear non-adherence (e.g., "sometimes forgets," "takes when needed," "missed a few doses").  • Clear adherence: The patient reliably takes medication as prescribed, demonstrates consistent routines, completes refills on time, and no clinician concerns are noted.    Assign a score from the following Likert scale:  • 3 = Definitely Non-Adherent  • 2 = Very Probably Non-Adherent  • 1 = Probably Non-Adherent  • 0 = Possibly Non-Adherent  • −1 = Probably Adherent  • −2 = Very Probably Adherent  • −3 = Definitely Adherent    Additional requirements:  • If the note is ambiguous, use contextual clues to assign your best estimate  • Ignore information unrelated to medication use unless it directly affects adherence (e.g., side effects, access issues)  • Protected health information (PHI) has been replaced with placeholders such as [**NAME**], [**AGE**], [**MEDICATION**], [**DATE**]  • Always assign exactly one Likert score |
| ***Output Format***  **Mode = Likert:**  Return only JSON: {"likert_score": <integer in [given scale]>, "likert_score_analysis": <one short sentence>}    **Mode = Binary:**  Return only JSON: {"label": "<Yes or No>"}    *Do not include any extra text, explanations, markdown, or code fences.* | **Mode = Likert:**  Return only JSON with exactly these keys:  { "likert_score": −3 \| −2 \| −1 \| 0 \| 1 \| 2 \| 3,  "likert_score_analysis": <one short sentence> }    **Mode = Binary:**  Return only this JSON exactly:  {"label": "Yes"} or {"label": "No"}    *Do not include any extra text, explanations, markdown, or code fences.* |

**Note.** MAB = Medication Adherence Behavior; PHI = Protected Health Information; NSSI = Non-Suicidal Self-Injury; SI = Suicidal Ideation. Italicized angle-bracket terms in the left column denote generalizable template placeholders. JSON output keys are shown verbatim as passed to the model. Positive Likert scores indicate non-adherence; negative scores indicate adherence.
